# The Value of Molecular vs. Morphometric and Acoustic Information for Species Identification Using Sympatric Molossid Bats

**DOI:** 10.1371/journal.pone.0150780

**Published:** 2016-03-04

**Authors:** Yann Gager, Emilia Tarland, Dietmar Lieckfeldt, Matthieu Ménage, Fidel Botero-Castro, Stephen J. Rossiter, Robert H. S. Kraus, Arne Ludwig, Dina K. N. Dechmann

**Affiliations:** 1 Department of Migration and Immuno-Ecology, Max Planck Institute for Ornithology, Radolfzell, Germany; 2 Department of Biology, University of Konstanz, Konstanz, Germany; 3 International Max Planck Research School for Organismal Biology, University of Konstanz, Konstanz, Germany; 4 Swedish University of Agricultural Sciences, Department of Animal Breeding and Genetics, Uppsala, Sweden; 5 Department of Evolutionary Genetics, Leibniz-Institute of Zoo and Wildlife Research, Berlin, Germany; 6 Institut des Sciences de l’Evolution, UMR 5554-CNRS-IRD, Université de Montpellier, Montpellier, France; 7 School of Biological and Chemical Sciences, Queen Mary University of London, London, United Kingdom; 8 Smithsonian Tropical Research Institute, Panamá, Rep. of Panamá; Università degli Studi di Napoli Federico II, ITALY

## Abstract

A fundamental condition for any work with free-ranging animals is correct species identification. However, in case of bats, information on local species assemblies is frequently limited especially in regions with high biodiversity such as the Neotropics. The bat genus *Molossus* is a typical example of this, with morphologically similar species often occurring in sympatry. We used a multi-method approach based on molecular, morphometric and acoustic information collected from 962 individuals of *Molossus bondae*, *M*. *coibensis*, and *M*. *molossus* captured in Panama. We distinguished *M*. *bondae* based on size and pelage coloration. We identified two robust species clusters composed of *M*. *molossus* and *M*. *coibensis* based on 18 microsatellite markers but also on a more stringently determined set of four markers. Phylogenetic reconstructions using the mitochondrial gene co1 (DNA barcode) were used to diagnose these microsatellite clusters as *M*. *molossus* and *M*. *coibensis*. To differentiate species, morphological information was only reliable when forearm length and body mass were combined in a linear discriminant function (95.9% correctly identified individuals). When looking in more detail at *M*. *molossus* and *M*. *coibensis*, only four out of 13 wing parameters were informative for species differentiation, with *M*. *coibensis* showing lower values for hand wing area and hand wing length and higher values for wing loading. Acoustic recordings after release required categorization of calls into types, yielding only two informative subsets: approach calls and two-toned search calls. Our data emphasizes the importance of combining morphological traits and independent genetic data to inform the best choice and combination of discriminatory information used in the field. Because parameters can vary geographically, the multi-method approach may need to be adjusted to local species assemblies and populations to be entirely informative.

## Introduction

Molecular biology, with the study of mitochondrial and nuclear genomes, has revolutionized our understanding of the distribution and evolutionary history of worldwide species diversity. In the context of mammalian species diversity, the order Chiroptera (bats) constitutes an exceptional taxon, with over 1331 species listed in a recent systematic review [1] representing a fifth of all extant mammals. Molecular studies have also led to the discovery of many cryptic lineages and boosted the number of described bat species. For example, analyses of mitochondrial genes revealed several cryptic species in well-studied areas such as Europe [2–6]. The use of DNA barcoding [7] led to a reevaluation of the number of bat species in the tropics [8–10]. Based on their sequence similarity, the barcodes can be clustered into a Molecular Operational Taxonomic Unit (MOTU) [11]. One great advantage of DNA barcoding is the important database available for comparative purposes (BOLD: The Barcode of Life Data System, [12]). However, DNA barcodes present pitfalls linked to maternal inheritance (reviewed in [13]) and should always be considered in conjunction with other sources of data. For instance, nuclear microsatellite loci were used successfully to identify *Pipistrellus kuhlii* as one biological species with two mitochondrial barcodes [14]. The use of nuclear microsatellites is also powerful to detect potential interspecific hybridization, otherwise undetected via the sole use of mitochondrial barcodes [15]. Other taxonomic parameters, such as morphological characters or echolocation calls, should also be combined with molecular data, following, for example, the framework of Integrated Operational Taxonomic Units (IOTUs) [16]. Integrating traditional taxonomy to molecular taxonomy is seen as the future of taxonomy [17].

Despite this recent boost of bat diversity with molecular species identification, the status of many bat taxa is not yet firmly established. The bat genus *Molossus* (family Molossidae; E. Geoffroy, 1805) is a typical example of this. These Neotropical bats occur from Northern Mexico to Southern Argentina. A systematic review from 1913 described a total of 19 species [18]. Many of these species were later synonymized and seven or eight species, depending on the authors, were recognized in the latest taxonomic reviews [19–22]. In addition, one species, *M*. *alvarezi*, was newly described based on size, pelage coloration and morphological characteristics [23]. Despite broad agreement among systematic reviews, the taxonomic boundaries and names within the genus are not settled. For example, *M*. *bondae* (J.A. Allen, 1904) and *M*. *currentium* (O. Thomas, 1901) can be grouped under the name *M*. *currentium* [19] or considered as two species based on their distribution in Central or South America [22]. Similarly, *M*. *molossus* (Pallas, 1766) has been described as being “desperately in need of revision” [19] and probably represents a species complex; indeed, *M*. *coibensis* (J. A. Allen, 1904) was treated as a synonym of *M*. *molossus* [24,25] yet is now considered a full species based on recent systematic assessments [19,21,22].

To date, few studies have applied molecular information to address questions regarding the taxonomy of the genus *Molossus*. The first molecular investigation of the evolutionary relationships within the genus relied on allozymes [21]. A more recent study identified only higher-level relationships between genera of the family Molossidae using one mitochondrial gene and three nuclear genes [26]. Here we compare molecular data from DNA-based markers with more commonly used morphometric and bioacoustic information to assess the reliability of each type of information for the identification of several *Molossus* species in Panama. We distinguished the *Molossus* species at our study site with a set of newly developed microsatellite markers, sequence data from the mitochondrial gene *co1* (DNA barcode), and the mitochondrial region d-loop for *M*. *molossus* and matched them with common field identification methods, i.e. morphological measurements and echolocation call recordings. While we were able to identify the molossid species at our site in Panama with our methods, we also find that one or even several field-based methods may not be sufficient for the proper identification of morphologically similar species whose traits may locally vary quite substantially.

## Materials and Methods

### Ethics statements

Capture and handling of animals was carried out with permission from the Autoridad Nacional del Ambiente in Panama with approval from the Institutional Animal Care and Use Committee of the Smithsonian Tropical Research Institute (2012-0505-2015). All animals were gently handled during measurements of morphological parameters, photographs of wings, genetic sampling and acoustic recording. All animals were released back in clearings in the same area in which they were captured. Heart tissue for genetic marker development came from a freshly deceased bat found in a private home.

### Sampling and data acquisition

During different fieldwork seasons between 2008 and 2013, we captured a total of 962 bats of the genus *Molossus* in Panama. Of these, 935 individuals were captured from various buildings in the village of Gamboa (Panama, 09°07’ N, 79°41’ W), 21 from the roof of the Smithsonian Tropical Research Institute’s (STRI) laboratory building on Barro Colorado Island (09°09’ N, 79°50’ W) as well as a dead tree off the shore of BCI, and seven from the roof of STRI’s dormitory at the Bocas del Toro research station (09°21’ N, 82°15’ W). We used mist-nets (Ecotone, Gdynia, Poland) to catch bats during their evening emergence. We determined sex, age, forearm length, body mass, reproductive status and marked each individual with unique subcutaneous passive integrated transponder (Trovan ID-100, Euro ID, Weilerswist, Germany). We also sampled wing membrane tissue using a biopsy punch (2 or 3 mm, Stiefel, U.S.A.) for genotyping purposes [27]. During some fieldwork seasons, we also collected wing photos and echolocation calls for some individuals. We selected data only for individuals that were genotyped later for microsatellites. We retained size-referenced wing photos for the 116 genotyped bats to obtain measurements for several wing parameters (see below for details on wing morphology evaluation). Finally, we selected echolocation calls for 80 genotyped bats. The recording protocol was as follows: bats were placed individually in a semi-open environment on a cloth wrapped over the end of a 2-meter pole to allow them to orientate and choose their moment of take-off freely. When the bat left the pole, acoustic recordings were made at a sampling rate of 448 kHz with an Acer Aspire One laptop computer (model KAV60, Acer Inc., Taiwan) using the Avisoft-UltraSoundGate 116H and the Avisoft-RECORDER USHG software (Avisoft Bioacoustics, Germany). Recordings were semi-automatic, with manual activation, a pre-trigger of 2 seconds and a post-trigger of 5 seconds to ensure the acquisition of full call sequences. The condenser microphone CM16/CMPA used (Avisoft Bioacoustics, Germany) had a sensitivity ranging from 10 to 200 kHz. The datasets of wing and echolocation calls overlapped for 35% of the analyzed individuals. The overlap of the datasets in terms of individuals is of minor concern here. We used microsatellite clusters (see further methods) to identify species for the individuals found in the different datasets. Our approach benefited from larger sample sizes that are more representative of the populations studied.

### Molecular analyses and species identification

A subset of the captured individuals (n = 27) was clearly identified as *M*. *bondae* based on their size and darker pelage coloration [22]. The species status of these 27 individuals was therefore not checked with molecular methods. For the remaining 935 individuals of *M*. *molossus* and *M*. *coibensis*, we used molecular methods; specifically i) for genetic clustering of nuclear microsatellite markers and ii) for phylogenetic tree reconstruction using 659 base pairs of the mitochondrial gene cytochrome oxidase subunit 1 (*co1*) and 615 base pairs of the hyper variable fragment of the control region (d-loop). Laboratory work with these markers was initially targeted at different questions, i.e. a study of genetic population structure in *M*. *molossus* as well as an exploration of fur color variation. This explains the use of different markers as well as protocols and number of individuals in each analysis.

#### Microsatellite development and genotyping

The detailed laboratory protocol for the nuclear microsatellite markers is available in the S1 Text. Eighteen primer pairs successfully amplified; we report the sequences, accession numbers for the NCBI Probe database, the fluorescent dyes and the multiplex combinations in S1 Table. We used these 18 microsatellite markers to genotype 935 individuals.

#### Microsatellite evaluation and clustering

To identify the number of species captured, we performed microsatellite-based clustering of 935 genotyped individuals. This aim was achieved in three steps: i) genetic clustering of individuals based on the 18 microsatellite loci, ii) assertion of different assumptions for the genetic analysis software packages used in this study (Hardy-Weinberg Equilibrium, low frequency of null alleles and linkage equilibrium) and iii) genetic clustering based on a robust, filtered set of those loci that adhered closely to the respective genetic assumptions. We first determined the number of genetic clusters corresponding to the number of species (at least two). We used the 18 microsatellite loci using a two-step Discriminant Analysis of Principal Components (DAPC [28]), a clustering method that does not require specific genetic assumptions for the loci used (unlike other clustering software that typically make use of patterns, e.g., Hardy Weinberg and linkage equilibria [28]). The second step consisted of checking three genetic assumptions within each cluster defined by DAPC: Hardy-Weinberg Equilibrium (HWE), low frequency of null alleles and linkage equilibrium. Only loci following these three conditions in each cluster were used for a second stringent clustering analysis performed using the software STRUCTURE 2.3.4 [29,30].

For the first part of the microsatellite analysis, we selected the number of genetic clusters (corresponding to the different species) based on Bayesian Information Criterion (BIC), a measure of the trade-off between goodness of fit and complexity of the model. We calculated the BIC for 18 clusters (the number of buildings sampled) and 100 PCs with the *adegenet* package [31] in R 3.1.0 (R Development Core Team 2014). A two-step Discriminant Analysis of Principal Components (DAPC) [28] was used to infer the selected number of clusters. We retained the number of principal component axes corresponding to ~80% of the cumulative score in the Principal Component Analysis step and the number of axis corresponding to the optimized *a*-score in the Discriminant Analysis step [32].

For each cluster defined with the DAPC, we identified the number of alleles at each locus, the heterozygosity (observed and expected), tested for deviations of HWE and estimated the null allele frequency using CERVUS v3.0.3 [33]. For each cluster, we also tested for linkage disequilibrium between all pairs of loci using the log likelihood ratio statistic and default parameters implemented in GENEPOP ON THE WEB [34,35] and we applied a Bonferroni correction to the significance level of 0.05 (0.05: 9 loci at HWE = 0.00556) to correct for multiple testing. For the following steps, we selected only loci that were in HWE, had an estimated null allele frequency < 0.10 with CERVUS, and were in linkage equilibrium for all clusters. It has recently been shown that null allele estimation with CERVUS can be misleading [36]. We therefore additionally used the software ML-NULL, which has been shown to perform best among a number of methods [36,37], to obtain additional estimates and confirm frequencies < 0.10. The outcomes of both methods (i.e., CERVUS and ML-NULL) did not differ in our case (results not shown).

The last clustering analyses were based on the selected number of genetic clusters in the data and only those loci closely following the genetic assumptions of HWE, null alleles and linkage equilibrium. As a complementary method to the two-step DAPC (following the procedure described earlier), we ran an analysis with the software STRUCTURE 2.3.4 [29,30]. We used default parameters from the software with an admixture model, a length of burn-in period of 20,000 and a number of MCMC repetitions after burn-in of 80,000. We performed 10 replicate runs for the number of determined genetic clusters and averaged the results in CLUMPP 1.1.2 [38]. A few individuals showed lower membership probability to a genetic cluster with STRUCTURE (< 0.9), even though showing a strong assignment with DAPC. We excluded these admixed individuals, potentially hybrids or attributed to the wrong species, to avoid potential mistakes in subsequent analyses. Indeed, it is known that DAPC can be over-confident in making genetic cluster assignments and more than one method should be utilized to check for cluster assignment [39]. The pruned dataset was used to identify the number of alleles for each cluster.

#### Sequencing and phylogenetic reconstructions

We sequenced *co1* for 96 individuals and d-loop for 150 individuals. The detailed laboratory protocol for the mitochondrial genes is available in the S1 Text. The newly generated sequences are available on GenBank, respectively under the accession numbers KT721362—KT721412 for the 51 *co1* sequences and KT721413—KT721441 and KT721443 –KT721563 for the 150 d-loop sequences.

We obtained 74 *co1* sequences from GenBank, including all sequences for *Molossus* and four outgroups from the molossid family (three species of *Cynomops* and one species of *Promops*). We aligned the 51 *co1* sequences from this study with the 74 GenBank sequences using MUSCLE [40] with default parameters as implemented in SeaView 4.5.4. [41]. We aligned the d-loop sequences from this study with MEGA 4.0 [42] and visually checked the alignment for repeated sequence arrays, a pattern already found in different bat species [43]. Three *Cynomops* species (GenBank accession numbers JF447634, JN312044 and EF080319) and *Promops centralis* (JF444936) were used as outgroups to root the tree inferred from *co1* sequences. The last sequence was labelled as *M*. *rufus* but we verified the species using the Barcode of Life Data System (more than 29000 sequences for the Order Chiroptera, [12]). The tree inferred from d-loop sequences was unrooted because we could not find publicly available sequences of close outgroups that could be satisfactorily aligned with our sequences. In order to find the best-fitting model for each gene, we compared 56 models of nucleotide evolution using jModelTest 2.1.7 and the Bayesian Information Criterion (BIC) [44]. The best-fitting model was then used in PAUP* 4b10 [45] to infer the respective phylogenies. Reliability of nodes was measured using 100 non-parametric bootstraps that were then mapped on the inferred trees using the *plotBS* option in the R package *phangorn* [46]. We validated the taxonomic identification of the sequences deposited in GenBank a posteriori (see discussion). The information on genetic clustering from the STRUCTURE analysis was also plotted on the tips of the final trees.

### Variation of fur color

We selected a set of eight individuals from the three species with pictures of fur color from the back of the individuals. This set of individuals was representative of the whole range of fur color observed in the field. This selection of pictures displayed the intra-species variation but also inter-species overlap in fur color. Our further use of the pictures to quantify colors was limited by the absence of camera calibration [47].

### Analyses of body parameters

We investigated morphological species differences based on two parameters: forearm length (mm) and body mass (g). We used these parameters to estimate a linear discriminant function using the “lda” function (library Mass) in R 3.1.0 [48] to separate the three *Molossus* species. Only adults, but not pregnant females, were used in the analysis. We calculated means and 95% confidence intervals (CI) for each combination of morphological parameter, species and sex. We obtain the 95% Confidence Intervals by using the formula provided by the R book [49]. We also assessed the classification rate of the species by the lda function with the leave-one-out cross-validation procedure.

### Analyses of wing shape

**Fig 1:**
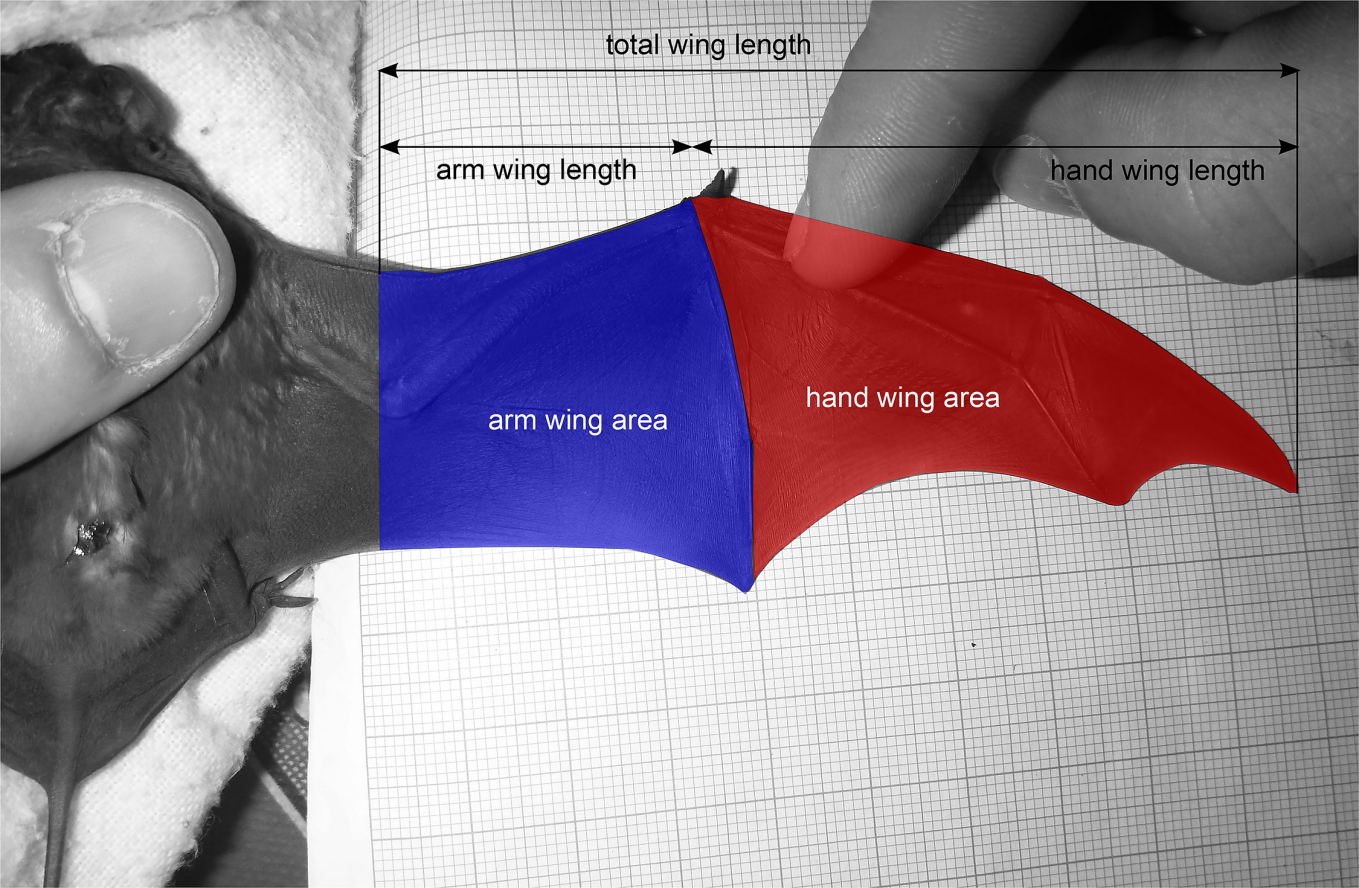
Right wing of a *Molossus molossus* showing areas used to analyze wing shape.

We used the wing photos to extract a series of wing parameters and morphological traits relevant to flight performance and foraging strategy [50]. We followed an established procedure to define landmarks and obtain the following measurements [51] from wing photos (Fig 1): forearm length (mm), total area (mm^2^), total wing length (mm), arm wing area (mm^2^), arm wing length (mm), hand wing area (mm^2^), hand wing length (mm), wing aspect ratio (wing length^2^/wing area), wing loading (body mass*g/wing area), tip length ratio (hand wing length / arm wing length), tip area ratio (hand wing area / arm wing area), tip shape index (tip area ratio / tip length ratio—tip area ratio) and a circularity index (4*π*wing area / wing perimeter^2^). In order to minimize inter-observer error, all measurements were collected by a single individual observer. We calculated the mean and the 95% CI [49] for each combination of wing parameter, species and sex.

### Analyses of echolocation calls

We analyzed echolocation calls from a subset of individuals genetically identified as *M*. *molossus* or *M*. *coibensis* using the software Batsound 4.1.300 (Pettersson Electronik AB, Uppsala, Sweden). We randomly selected sequences of up to ten calls that contained a sufficient signal to noise ratio for each individual. Sampling frequency was configured at 44.1 kHz, with 16 bits per sample and a 512-point FFT with a Hamming window for analysis. A 112 Hz frequency resolution was obtained for spectrograms and power spectrum. In each call, we measured six echolocation parameters using the software Batsound (Pettersson Elektronik AB, Sweden). From the spectrogram, based on the fundamental call, we measured 1) the Start Frequency (SF; frequency measured at the beginning of the call), 2) the End Frequency (EF; frequency measured at the end of the call) and 3) the bandwidth (BW; difference between SF and EF) in kHz. From the maximal intensity in the power spectrum, we determined the 4) Peak Frequency (PF). From the oscillogram, we extracted 5) the Duration (D) and 6) the Pulse Interval (PI; time interval between two consecutive calls) in ms.

**Fig 2:**
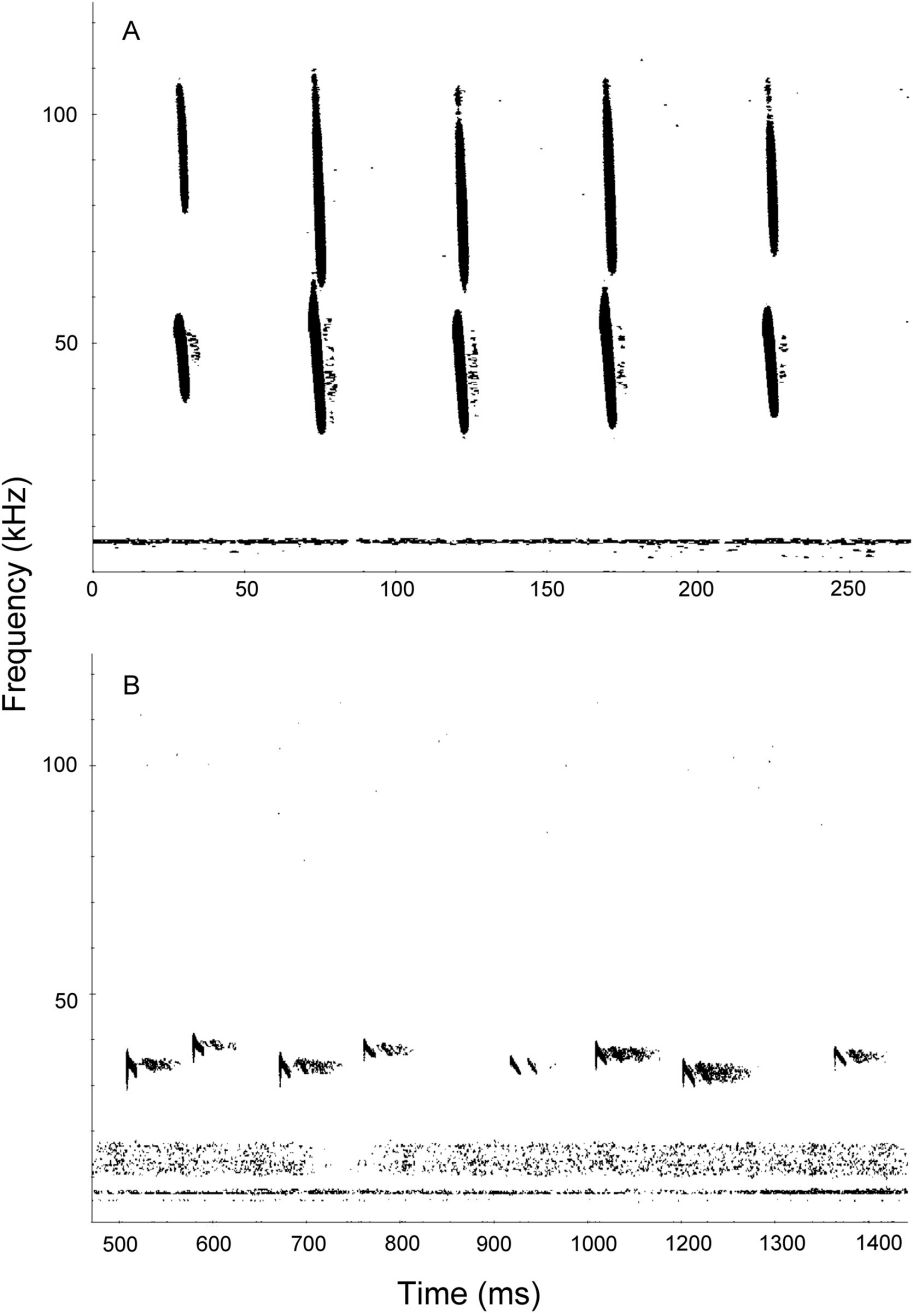
Sonograms of the two types of calls informative for identification found in *Molossus molossus* and *coibensis*. (A) Approach calls with harmonics. (B) Search calls alternating a lower and higher frequency pulse (respectively SI and SII).

First, we analyzed all calls to examine the entire recorded acoustic diversity. We found a great range of variability in the calls, consistent with previous studies on *M*. *molossus* in Belize and Cuba [52,53]. We examined the Pearson’s product moment correlation using R 3.1.0 [48]. Only two of the acoustic parameters (SF and PF) showed a strong correlation of 0.95 (all others ranging from -0.69 to 0.85). We excluded PF and ran a Principal Component Analysis (PCA) of all calls with the five remaining acoustic parameters. Secondly, we categorized our different sequences of calls into call types. A typical sequence of calls started at the release perch with short calls with a downward frequency modulation and a prominent second harmonic, similar to the approach call recorded for *M*. *molossus* in the vicinity of their roosts in Cuba [54]. We also recorded search flight calls with narrow bandwidths [54] when a bat was higher above the ground. Search flight calls were typically two-toned and alternating between a lower frequency pulse (SI) and a higher frequency pulse (SII) [53,54]. Some search flight calls were also irregularly alternating the SI and SII or were three-toned, a known pattern for this species [55,56]. For our purpose of species comparisons, we selected only sequences with a clear call structure: the approach calls where all calls had harmonics and the two-toned search flight calls consistently alternated with a lower and higher frequency pulse (SI and SII, Fig 2). For each combination of call type and species, we calculated mean and the 95% CI [49]. We disregarded sequences of calls that could not be firmly categorized such as sequences of approach calls that did not always show harmonics, sequences mixing approach calls and search flight calls as well as search flight calls irregularly alternating the tones or showing an uncertain number of tones.

## Results

### Microsatellite evaluation and clustering

We genotyped 935 individuals at 18 microsatellite loci (for dataset, see S1 Dataset). Based on the complete dataset with 18 loci, we selected *K* = 2 clusters because of the shape of the BIC curve as a function of the number of clusters (ranging from one to 18), showing a much better likelihood for *K* = 2 than for *K* = 1 and only little gain in likelihood for additional clusters (S1 Fig). In the two-step DAPC, we retained 60 axes (~80% of the cumulative variance) in the Principal Component Analysis step and one axis (optimized *a*-score) in the Discriminant Analysis step. From the 935 individuals, 841 were attributed to *Cluster One* and 94 to *Cluster Two*.

For these genetic clusters, we list the number of alleles, the range of allele size, the observed and expected heterozygosities and the estimated null allele frequency in Table 1. Two loci from *Cluster One* and three from *Cluster Two* significantly departed from Hardy-Weinberg equilibrium (HWE). Three loci from *Cluster One* and six from *Cluster Two* showed high estimated null allele frequencies (over 10%). Two of the loci from *Cluster Two* departing from the HWE also had high estimated null allele frequency, potentially resulting from null amplification. Of the nine loci at HWE, many pairs showed significant linkage disequilibrium (22 for *Cluster One* and nine for *Cluster Two* out of 36). The only loci in HWE, in linkage equilibrium and with estimated null allele frequency < 0.10 across the two clusters were C56, C77, C115 and C132.

**Table 1:** Cross-amplification and genetic tests for 18 *Molossus molossus* loci grouped in two genetic clusters. The columns respectively represent: A, Number of alleles; AS, range of allele sizes (bp); Ho, observed heterozygosity and He, expected heterozygosity; F(null), estimated null allele frequency. Loci or values highlighted in boldface departed significantly from HWE (following p-values from testing in CERVUS) or had high estimated null allele frequencies (> 0.10 in CERVUS).

Genetic cluster	*Cluster One* (n = 841)				*Cluster Two* (n = 94)			
Locus	A	AS (bp)	Ho / He	F(null)	A	AS (bp)	Ho / He	F(null)
Mol_A2	9	191–211	0.74 / 0.76	0.02	7	189–203	0.65 / 0.68	0.01
**Mol_A221**	11	286–321	0.45 / 0.47	0.02	7	286–315	0.14 / 0.18	**0.16**
**Mol_B233**	4	198–209	0.51 / 0.54	0.02	3	198–205	0.02 / 0.08	**0.48**
Mol_C3	11	257–284	0.81 / 0.80	-0.01	11	261–286	0.82 / 0.8	-0.02
**Mol_C6**	7	101–117	0.52 / 0.60	0.06	6	100–107	**0.16 / 0.53**	**0.55**
Mol_C20	4	136–142	0.71 / 0.72	0.00	5	143–151	0.58 / 0.59	0.00
Mol_C27	23	270–320	0.83 / 0.87	0.02	7	268–304	0.33 / 0.40	0.09
Mol_C56	18	171–213	0.88 / 0.84	-0.02	12	186–210	0.83 / 0.84	0.00
**Mol_C61**	19	177–214	**0.78 / 0.89**	0.07	6	182–198	0.59 / 0.61	0.01
Mol_C77	11	198–225	0.71 / 0.73	0.02	5	198–231	0.27 / 0.30	0.05
**Mol_C109**	17	255–293	0.60 / 0.87	**0.18**	7	243–277	0.31 / 0.79	**0.44**
**Mol_C109bis**	18	218–247	**0.81 / 0.83**	0.01	6	214–226	0.61 / 0.63	0.02
Mol_C114	12	268–310	0.78 / 0.76	-0.02	10	268–313	0.73 / 0.81	0.05
Mol_C115	12	265–294	0.75 / 0.78	0.02	7	265–282	0.61 / 0.67	0.05
**Mol_C117**	15	294–348	0.28 / 0.85	**0.51**	6	298–340	0.57 / 0.55	-0.01
**Mol_C118**	10	213–224	0.55 / 0.81	**0.19**	6	214–224	**0.06 / 0.51**	**0.81**
Mol_C132	15	147–182	0.77 / 0.81	0.03	4	178–186	0.42 / 0.46	0.03
**Mol_D109**	20	291–324	0.86 / 0.90	0.02	6	296–317	0.03 / 0.07	**0.42**

**Fig 3:**
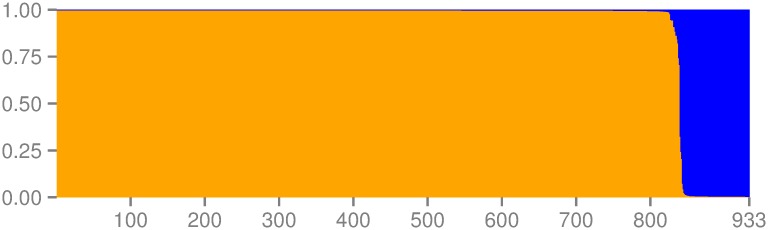
Genetic clustering (*K* = 2) of 933 *Molossus* bats from the village of Gamboa. *Cluster One* is represented in orange and *Cluster Two* in blue. The two clusters were obtained with four microsatellite primers using the software STRUCTURE. The few individuals at the edge of the two clusters are admixed.

We consequently based all following clustering analyses on only four loci and two clusters. We also excluded two individuals with missing data for these specific loci. Some individuals retained also showed missing data at two loci (n = 6) and one locus (n = 87) of these four. The performance of the DAPC and STRUCTURE analyses on four loci matched that of the analyses with 18 loci resulting in two similar genetic clusters. The majority of individuals were clearly found in *Cluster One* and *Cluster Two* (respectively orange and blue on Fig 3). Only ten individuals out of 933 (1%) showed discrepancies in clustering, with clear assignment to a cluster in the DAPC but admixture in the STRUCTURE analysis (posterior assignment probability < 0.9). These individuals with uncertain assignment were removed from the subsequent analyses as explained in the methods. The pruned dataset was composed of 923 individuals: 833 in *Cluster One* that occurred in all 18 sampled buildings and 90 in *Cluster Two* found in five of the 18 sampled buildings. These two clusters were used to determine the number of alleles for each of the 18 loci (S2 Table).

### Phylogenetic results: the *co1* tree

The final co1 alignment consisted of 659 nucleotides for 125 individuals (S2 Dataset). Phylogenetic reconstruction under a Tamura-Nei (TrN) model with rate variation among sites (Γ) and a proportion of invariable sites (I) displayed numerous polytomies with most nodes showing low statistical support (<70%) (S2 Fig). The majority of samples genotyped with co1 (96.1%) were also genotyped using microsatellites, the membership to the microsatellite clusters are represented in color at the nodes of the tree. In the *Molossus* crown, bootstrap values were >70% in only five nodes. The tree consisted mostly of i) deep rooted clades comprising newly sampled *M*. *molossus* from Panama together with published haplotypes from Ecuador, Suriname and Guyana, ii) a “floating” clade from Panama and iii) a tree crown with two *M*. *rufus*, four *M*. *coibensis* and a few more individuals from Panama. The tree crown was composed of a well-supported clade with two *M*. *rufus* (BS = 100) and a polytomy composed of a *M*. *sp*. from Venezuela (JF447833), a clade with four *M*. *coibensis* and a *Molossus sp*. (JF442201) from Ecuador and a clade with 11 of our individuals from Panama. As these 11 bats were also found in *Cluster Two* from the microsatellite clustering analysis, we later considered all these animals to be *M*. *coibensis*. The deeper branches of the tree consisted of *M*. *molossus* from Panama, Ecuador, Suriname and Guyana. At the roots of the tree, the 22 individuals from Panama were mixed together with the GenBank sequences of *M*. *molossus*, with no clear biogeographic pattern. We also found a well-supported clade with 17 bats from Panama (BS = 98), sister to the crown tree. However, the 22 individuals mixed with the GenBank sequences and the 17 individuals from this Panamanian clade were previously grouped in the same *Cluster One* from the microsatellite clustering analysis. We therefore did not consider the Panamanian clade as a new species, but rather a “floating” clade and defined all individuals from *Cluster One* as *M*. *molossus* in this tree and subsequent analyses.

### Phylogenetic results: the d-loop tree

The final alignment of d-loop was 615 base pair long and encompassed 150 individuals (S3 Dataset). The 150 new d-loop sequences showed high genetic diversity, with 42 different haplotypes. Most haplotypes (n = 28) were found in one individual, except for a common haplotype that was shared by 38 individuals. Fourteen haplotypes were shared between different roosts, one of them being shared between ten roosts. According to the BIC criterion, the Hasegawa-Kishino-Yano model (HKY) with a proportion of invariable sites (I) was the best fitting model for the tree reconstruction (S3 Fig). The d-loop tree presented a similar topology to the *co1* tree, with several clades of *M*. *molossus* and a clade with *M*. *coibensis*. One of the individuals from this tree (KT721428) was previously identified as *M*. *coibensis* in the *co1* tree (KT721364, transponder number EAC87), we therefore assigned its clade in the d-loop tree to the species *M*. *coibensis* and the rest of the individuals to *M*. *molossus*. Statistical support for the *M*. *coibensis*’ clade and six subclades of *M*. *molossus* was high (BS ≥ 90). Most of the d-loop sequences (77.3%) were also genotyped for microsatellites, the membership to the microsatellite clusters are represented in color at the nodes of the tree.

### Variation of fur color

**Fig 4:**
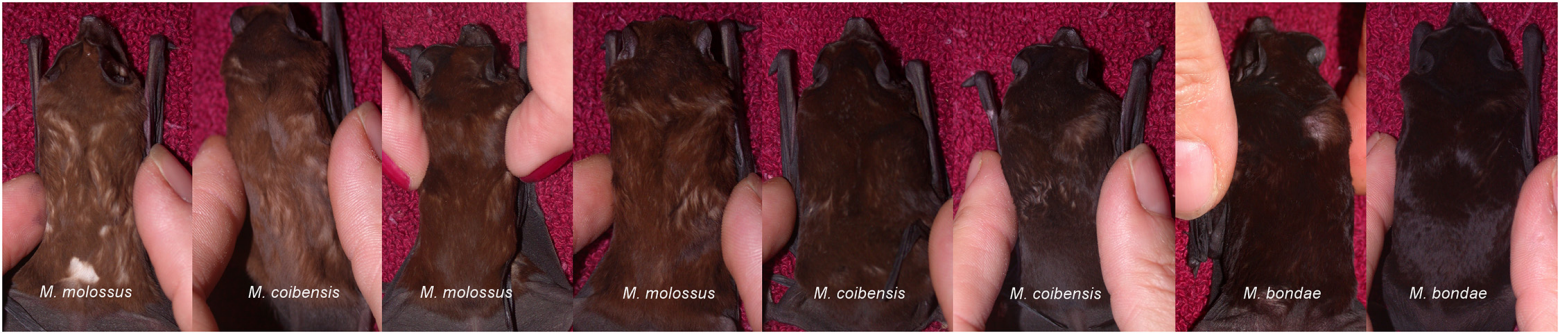
Variation of fur color (back) for eight individuals of *M*. *molossus*, *M*. *coibensis* and *M*. *bondae* from Panama.

The subset was composed of two *M*. *bondae* identified morphologically as well as three *M*. *molossu*s and three *M*. *coibensis* confirmed genetically. We observed a fur color on the back ranging from light brown to dark brown. The similarity of the fur color emerges clearly from this panel of three species (Fig 4).

### Analyses of body parameters

**Fig 5:**
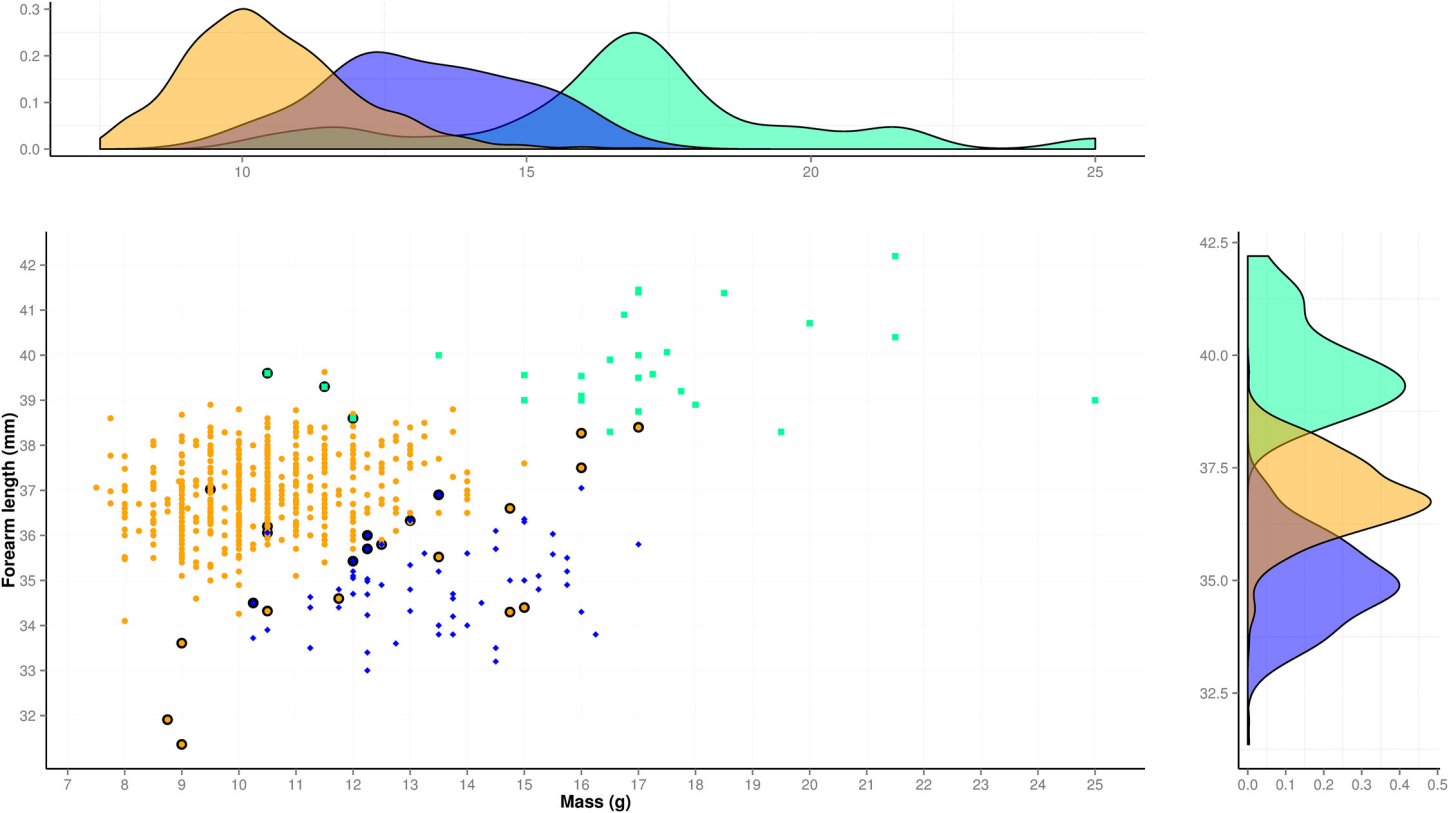
Forearm length (mm) plotted against body mass (g) for three Panamanian *Molossus* species. The color code is as follows: *M*. *molossus* (orange dots), *M*. *coibensis* (blue diamonds) and *M*. *bondae* (green squares). Following the same color code, the frequency distribution of body mass is plotted above the graph and the frequency distribution of forearm length on the right side of the graph. Points outlined in black are misclassified individuals based on the linear discriminant function and the leave-one-out cross-validation procedure (4.1% of the individuals).

We obtained morphological data from 617 adults of both sexes: 526 *M*. *molossus*, 64 *M*. *coibensis* and 27 *M*. *bondae* (the first two genetically assigned to species; S4 Dataset). Forearm length (mm) and body mass (g) were normally distributed and overlapped between the three species (Fig 5). Forearm length was ranked in increasing order for *M*. *coibensis*, *M*. *molossus* and *M*. *bondae*. Body mass was ranked in increasing order for *M*. *molossus*, *M*. *coibensis* and *M*. *bondae*. Only *M*. *molossus* showed body mass below 9.5 g while only *M*. *bondae* had forearm length above 39.63 mm and body mass above 17 g. Twenty-five of the 617 bats (4.1%) were misclassified by the lda function using forearm length and body mass. Misclassification occurred for animals with extreme values for the species range. Thus, the lightest *M*. *bondae* (n = 3, range = 10.5–12.0 g) and the heaviest *M*. *molossus* (n = 3, range = 16.0–17.0 g) were wrongly identified as well the smallest *M*. *molossus* (n = 9, range = 31.6–36.6 mm) and the largest *M*. *coibensis* (n = 10, range = 34.5–37.02 mm).

We provide means, 95% CI and range of forearm length and body mass in Table 2 for each combination of species and sex. We found a significant male-biased sexual dimorphism in all three species at the intra-specific level, as the 95% CI did not overlap. Inter-specific values differed significantly for both parameters based on the 95% CI.

**Table 2:** Sex-specific means, [95% CI], sample size and range of forearm length and body mass.

Parameter	Forearm length			Body mass (g)		
Sex	Female		Male	Female		Male
*M*. *coibensis*	34.6	<	35.6	12.8	<	14.1
	[34.3–34.8]		[35.3–35.9]	[12.3–13.3]		[13.5–14.8]
	(n = 41, 33.0–37.0)		(n = 23, 34.0–37.0)	(n = 41, 9.5–16.2)		(n = 23, 11.7–17.0)
*M*. *molossus*	36.7	<	37.4	10.2	<	11.4
	[36.6–36.8]		[37.2–37.6]	[10.1–10.3]		[11.1–11.6]
	(n = 403, 31.9–38.8)		(n = 123, 31.4–39.6)	(n = 403, 7.5–16.0)		(n = 123, 7.7–17.0)
*M*. *bondae*	39.4	<	40.6	16.4	<	17.9
	[39.0–39.8]		[39.8–41.3]	[14.9–18.0]		[15.8–20.0]
	(n = 18, 38.3–41.4)		(n = 9, 39.0–42.2)	(n = 18, 10.5–25.0)		(n = 9, 13.5–21.5)

Based on the leave-one-out cross-validation procedure, the overall classification rate of the function was 95.9% (97.7% for *M*. *molossus*, 84.4% for *M*. *coibensis* and 88.9% for *M*. *bondae*). Only 25 of 617 individuals (4.1%) were misclassified based on the combined two morphological parameters alone (symbols outlined in black in Fig 4).

### Analyses of wing shape

We analyzed wing photos (S5 Dataset) of 104 *M*. *molossus* (87 females and 17 males) and 12 *M*. *coibensis* (8 females and 4 females). The means and 95% CI for each combination of wing parameter, species and sex are summarized in Table 3. Four parameters were significantly different between species: *Molossus molossus* had longer forearms (confirming the results outlined in the previous paragraph), larger hand wing area, and a longer hand wing, while wing loading was greater in *M*. *coibensis*. Wrongly classified by the lda function as *M*. *molossus*, a female *M*. *coibensis* (E8472) had values for the wing parameters falling within the 95% CI of *M*. *molossus*.

**Table 3:** Mean [95% CI] of wing parameters for species and sex. The four parameters highlighted in bold differed significantly between species. For each species, the intermediate column compares mean values between sexes.

Species	*Molossus coibensis*			*Molossus molossus*		
Sex	Female (n = 8)		Male (n = 4)	Female (n = 87)		Male (n = 17)
**Forearm length (mm)**	33.8	<	35.3	36.7	<	37.1
	[32.5–35.1]		[35.0–35.6]	[36.6–36.9]		[36.7–37.5]
Total area (mm²)	2292	<	2469	2792.1	>	2660.6
	[2064–2520]		[2233–2706.0]	[2739–2845]		[2531–2789]
Total wing length (mm)	94.8	<	99.7	103.8	>	102.7
	[89.3–100.2]		[92.5–107.0]	[102.7–104.9]		[100.1–105.3]
Arm wing area (mm²)	1086	<	1263	1354	>	1321
	[939–1233]		[1103–1422]	[1319–1388]		[1226–1416]
Arm wing length (mm)	35.1	<	39.3	38.8	>	38.5
	[32.0–38.2]		[34.4–44.2]	[38.3–39.4]		[36.9–40.1]
**Hand wing area (mm²)**	1179	<	1211	1432	>	1361
	[1092–1266]		[1107–1315]	[1404–1459]		[1318–1403]
**Hand wing length (mm)**	59.9	<	60.0	64.9	>	64.1
	[57.2–62.5]		[57.6–62.3]	[64.0–65.7]		[62.6–65.6]
Circularity	0.49	>	0.48	0.51	>	0.49
	[0.48–0.51]		[0.47–0.49]	[0.50–0.51]		[0.48–0.50]
Tip area ratio	1.1	>	1.0	1.1	>	1.0
	[1.0–1.2]		[0.8–1.1]	[1.0–1.1]		[1.0–1.1]
Tip length ratio	1.7	>	1.5	1.7	=	1.7
	[1.6–1.8]		[1.4–1.7]	[1.6–1.7]		[1.6–1.7]
Tip shape index	1.8	>	1.7	1.9	>	1.7
	[1.5–2.1]		[1.1–2.3]	[1.7–2.0]		[1.5–1.9]
Aspect ratio	3.9	<	4.0	3.9	<	4.0
	[3.8–4.1]		[3.8–4.3]	[3.8–3.9]		[3.9–4.1]
**Wing loading (Nm**^-^**²)**	53.9	<	56.1	39.3	<	43.2
	[46.9–60.9]		[47.6–64.5]	[37.9–40.7]		[40.6–45.9]

### Analyses of echolocation calls

**Fig 6:**
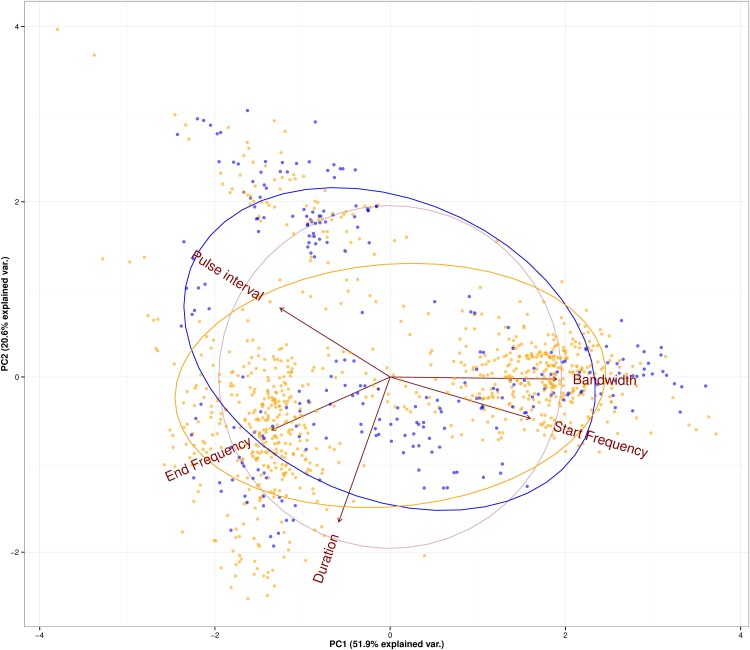
Principal Component Analysis of five acoustic parameters for *M*. *molossus* (orange) and *M*. *coibensis* (blue).

We recorded echolocation calls of 80 adult bats: 65 *M*. *molossus* and 15 *M*. *coibensis*. We measured 8 to 30 calls per individual, resulting in 834 for *M*. *molossus* and 255 pulses for *M*. *coibensis* (S6 Dataset). When analyzing unclassified calls, we found no clear species differences based on the Principal Component Analysis (Fig 6), with just 51.9% of the variance explained by the first axis and 21.6% by the second axis.

We found sequences of approach calls with harmonics in 35.4% of the *M*. *molossus* and 46.7% of the *M*. *coibensis*. We observed search calls that regularly alternated between the two tones SI and SII in 18.5% of the *M*. *molossus* and 40.0% of the *M*. *coibensis*. Average SF and EF were higher in approach calls of *M*. *coibensis* but lower in the two-toned calls. For the two-toned calls, call duration was shorter in *M*. *molossus*, and bandwidth was higher in *M*. *coibensis*. Finally, pulse interval in the SII of the search calls was higher in *M*. *coibensis*. Mean values and SD for the five acoustic parameters and the different call types are compiled in Table 4 (for results of t-tests, see S3 Table). None of the individuals misclassified by the lda function had categorized calls to be compared to the table.

**Table 4:** Comparison of acoustic parameters between *M*. *molossus* and *M*. *coibensis*. Values are means *±* 1 standard deviation. Values in boldface represent significant differences between species based on a Student’s t-test for the given acoustic parameter. The two figures for sample size indicate the number of individuals and the number of calls.

Call type	Species	Sample size	Start Fr. (kHz)	End. Fr.(kHz)	Bandwidth (kHz)	Duration (ms)	Pulse interval (ms)
Approach call	*M*. *coibensis*	7 (70)	**56.1 ± 2.9**	**30.6 ± 5.5**	25.5 ± 6.4	0.4 ± 0.05	62.3 ± 54.9
	*M*. *molossus*	23 (226)	**52.8 ± 3.7**	**29.0 ± 3.2**	23.8 ± 4.9	0.4 ± 0.08	50.7 ± 37.3
Two-toned SI	*M*. *coibensis*	6 (29)	**35.4 ± 1.3**	**29.8 ± 1.9**	**5.6 ± 1.3**	**0.4 ± 0.4**	76.2 ± 23.6
	*M*. *molossus*	12 (60)	**39.1 ± 3.6**	**34.4 ± 3.8**	**4.7 ± 2.0**	**0.6 ± 0.3**	75.4 ± 24.6
Two-toned SII	*M*. *coibensis*	6 (29)	**39.7 ± 2.1**	**35.0 ± 1.8**	**4.7 ± 1.2**	**0.3 ± 0.3**	**153.8 ± 58.9**
	*M*. *molossus*	12 (60)	**42.8 ± 2.9**	**39.1 ± 3.0**	**3.8 ± 1.4**	**0.6 ± 0.3**	**117.6 ± 44.5**

## Discussion

Our combination of morphometric and molecular data confirmed the sympatry of at least three species: *Molossus molossus*, *M*. *coibensis* and *M*. *bondae* at our study site in Panama. *Molossus molossus* was more abundant than *M*. *coibensis* in the sampled buildings while *M*. *bondae* was rarely captured.

### Microsatellite clustering

Microsatellite analyses were useful for detecting species but also to reveal potential interspecific hybrids. Both the DAPC BIC method based on 18 loci and the STRUCTURE method based on four loci, recovered two genetic clusters, with consistent cluster membership with few exceptions. Our clustering analyses are therefore very robust to the choice of markers. Only 1% of the individuals clearly assigned to one cluster with the DAPC method showed admixture based on STRUCTURE. Sequencing a subset of these individuals with the gene *co1* revealed that the two microsatellite clusters corresponded to *M*. *molossus* and *M*. *coibensis*. As we successfully genotyped both species using microsatellites, they offer the potential for cross-species amplification in the genus *Molossus*. Multiple and non-exclusive explanations such as non-random mating could be an explanation as to why so many of the loci diverged from HWE and/or showed high prevalence of null alleles [57]. We may have sampled a non-random subset of the males in the gene pool [58]; in particular, our sampling was biased towards harem social groups occupying buildings [59] whereas unsampled males were probably solitary and roosting elsewhere. The four loci that stringently followed the genetic assumptions for the STRUCTURE analyses revealed a low number of admixed individuals (n = 10), representing only 1% of all genotyped individuals. Admixture signatures in STRUCTURE can result from interspecific hybridization or retention of ancestral polymorphism [15,60–62]. Admixture can also result from microsatellite size homoplasy, a well-described phenomenon that remains infrequently tested [63,64]. Further investigation of admixture in this study, be it technical artifact or biological reality, is not of relevance here because of its low incidence, and therefore out of scope presently. Further analysis will be required to identify the mechanism leading to admixture and the taxonomic status of admixed individuals.

### Phylogenetic reconstructions

Sympatry of very similar-looking species is common in bats [3,65,66] and can make species identification in the field extremely difficult. We successfully clustered individuals according to species with the *co1* sequences. In addition, the *co1* tree allowed us to incorporate GenBank sequences from different species and countries. We thus matched our sequences from Panama to *M*. *molossus* from Guyana, Ecuador and Suriname, and to *M*. *coibensis* from Ecuador. Our phylogenetic reconstructions provided the second piece of molecular evidence that *M*. *coibensis* and *M*. *molossus* occur in sympatry in Panama following the allozyme study by Dolan [21]. We found that the two species occur in the same buildings where they probably form species-specific social groups. Sympatric individuals of these two species have previously been reported for the province of Napo in Ecuador [9,67]. Our phylogenetic tree also revealed a “floating” clade of *M*. *molossus* that we were not able to match to GenBank sequences. With low statistical support (BS = 23), this phylogenetic uncertainty may represent a soft polytomy that could be resolved with increased character sampling [68]. The same 17 individuals were assigned to *M*. *molossus* in the microsatellite clustering analyses. This molecular differentiation may result from the occurrence of two distinct barcodes in the same species, as recently found in the bat *Pipistrellus kuhlii* [14]. The phylogenetic tree also revealed three GenBank sequences that were probably wrongly identified: JF442201 from Ecuador and JF447833 from Venezuela are probably not *M*. *molossus* but *M*. *coibensis*, and the inverse is true for JF442240 from Ecuador. Quality control of sequences using phylogenetic analyses [69] could easily avoid taxonomic misidentification of sequences submitted in public databases [70]. Despite its utility for species identification, our phylogenetic reconstructions using *co1* recovered a large polytomy with limited statistical support for the majority of nodes, and further phylogenetic reconstruction based on the fast-evolving mitochondrial region d-loop also recovered clades with low support in most cases. Future studies incorporating nuclear genes [68,71,72], combined datasets (i.e. morphology and genetics) [73] or even complete genomes [74–76] will thus be valuable in further elucidating the taxonomy of morphologically similar molossid bats. Whole genome analyses of thousands of species have been envisioned for many years [77] and this is becoming a reality with the development of next generation sequencing technologies, for example, birds with the B10K initiative [78,79].

### Variation of fur color

The panel of fur color (Fig 4) reveals the overlap between species, namely between *M*. *molossus* and *M*. *coibensis*.

### Analyses of body parameters

In contrast to the molecular methods, simple morphological parameters such as forearm length and body mass can easily be obtained in the field [80]. Used separately, these morphological parameters did not allow good discrimination of the three species due to the overlap in parameter distributions and the flip in ranks. Only when analyzed together in a linear discriminant function, the two parameters led to a high average rate of correct identification of the three species (95.9%). At the species level, the classification rate was ranked in decreasing order for *M*. *molossus* (97.7%), *M*. *bondae* (88.9%) and *M*. *coibensis* (84.4%). Similarly, for example, *Myotis* from Switzerland can be most reliably distinguished using a canonical discriminant function based on the forearm length and the ear length [81]. *Rhinolophus* from Bulgaria, Greece and Turkey can be correctly assigned using a canonical discriminant function of the forearm length and the first phalanx of the fourth finger [82] but for *Rhinolophus* from Tunisia the second phalanx of the fourth finger has to be added to the discriminant function [83]. The discriminant analysis constitutes a powerful approach to differentiate between morphologically similar species but only if the right combination of parameters from correctly assigned subsets of the individuals can be found. In addition to the species differences in body mass and forearm length, we also observed significant sexual size dimorphism with larger and heavier males in the three species. The sample size is low for *M*. *coibensis* (eight females and four males) but the reference values of the two parameters for each sex (Table 3) should allow correct species identification for most individuals of our three focal species in Panama.

### Analyses of wing shape

Wing shape is under strong selection for ecological niche use in bats because it underpins flight performance and foraging strategy [50]. Wing shape can also be useful for species identification [51,82]. However, all molossid bats have narrow wings well-adapted to hunting insects in open spaces [84] and, therefore, many of the wing parameters that we measured did not vary between species. We found higher values of forearm length (mm), hand wing area (mm²) and hand wing length (mm) in *M*. *molossus*, indicating longer wings at our study site. However, *M*. *coibensis* had higher wing loading (Nm^-^²), suggesting higher flight speed and turning agility than *M*. *molossus* [50]. While such variation may potentially be ecologically significant for niche separation between the two species, significant intraspecific geographic variation in wing parameters can be found, for example in rhinolophids [82]. Until a dataset from a broader geographic range is available, our values should only be used for comparisons with individuals within Central America or even only Panama.

### Analyses of echolocation calls

Acoustic recording of free-flying bats is a widespread method for surveys and species differentiation including different species of *Molossus* [52,55,85]. Acoustic recordings after release such as ours are also commonly used but not, to date, in molossids. The method is generally criticized as being not representative of the environment and associated calls in free-flying animals [86] but remains invaluable to match acoustic and molecular data of the same individual. Acoustic recordings after release proved useful only for a subset of our recordings after we categorized into different types of calls. The manual categorization of the calls confirmed the previously described call diversity: approach calls with harmonics described in *M*. *molossus* [53,54], two-toned search calls (described in the Molossidae and Vespertilionidae [87,88]) and three-toned search calls (*M*. *molossus* [55]). We only selected the two unmistakable categories: short calls with a downward frequency modulation and a prominent second harmonic (approach calls) and the alternating two-toned calls (search calls). Only a few call parameters showed significant differences between the two species, especially start and end frequency. Despite the apparent utility of these calls to discriminate species, several limitations to this method must be considered. For example, mean values and standard deviations for SI and SII strongly overlapped between *M*. *molossus and M*. *coibensis* (e.g. 0.3 ± 0.3 ms & 0.4 ± 0.4 ms vs. 0.6 ± 0.3 ms and 0.6 ± 0.3 ms). Following the recommendation of Barclay [89], our reference values should only be compared to individuals released under the open sky and away from background clutter.

### Comparison to other studies

Previous studies have reported reference values for different morphological parameters. However, several of these studies provided reference values using a different taxonomy from the one we used. For example, Simmons [19] considered *M*. *bondae* and *M*. *currentium* grouped under the name *M*. *currentium* and Reid [25] treated *M*. *coibensis* as a subspecies of *M*. *molossus*. Studies that followed the same taxonomy as we did provided values consistent with our results (based on molecular validation): for example, a range of 33.2–36.0 mm, 33.5–34.7 mm or 33.9–36.0 mm for *M*. *coibensis* [22,90,91], 35–40 mm for *M*. *molossus* [22] and 38.4–41.1 mm or 38–43 mm for *M*. *bondae* [22,25]. Measurements of body mass showed similar values too, with a range of 16–21 g for *M*. *bondae* [25]. However, larger values of forearm length in *M*. *coibensis* and *M*. *molossus* were found in a study from southeastern Brazil [92]. *M*. *coibensis* showed a range of 35.5–38.1 mm for females and 36.5–38.1 mm for males while *M*. *molossus* showed a range of 35.3–42.2 mm for females and 38.2–42.3 mm for males. The inconsistency between the values reported in our study in Panama and the one in Brazil could be a result of natural geographic variation—a common phenomenon in bats [93]—or from incorrect taxonomic attribution leading to wrong values in other studies. Problems in taxonomic attribution can result from unsettled taxonomy, for example *M*. *coibensis* from French Guyana referred to as a true species [22] or as the separate species *M*. *barnesi* [19,91]. To tackle these issues, additional comparative studies using molecular validation are required to provide reference values for these species in other regions of their biogeographic ranges.

## Conclusions

All methods we used, based on molecular, morphometric or acoustic data, provided useful information for species discrimination. However, all of these methods had their limitations too. While we were able to reliably identify *M*. *bondae* based on size and pelage coloration, the microsatellite analysis led to a reliable genetic clustering of *M*. *coibensis* and *M*. *molossus* using two different methods. Individuals were assigned correctly with just a few exceptions when using all 18 microsatellite loci as well as with the more stringently determined subset of markers (n = 4). However, developing microsatellite primers involves a considerable effort. Other molecular methods, PCR-based assays [94] or high resolution melting [95] are promising alternatives that should allow cheaper and faster results of similar quality. The phylogenetic reconstructions with the *co1* sequences were also useful for species identification but mitochondrial DNA markers alone did not provide strong clade support. Comparison of morphometric parameters, i.e. forearm length and body mass, was a simple and very useful tool for species discrimination. However, they were only discriminatory when combined in a linear discriminant analysis function or when the sex of the individuals was taken into account. Previous work on other species shows that a different trait combination may need to be found for each local species assembly, which may only allow correct species assignment after fieldwork has been completed, similar to the molecular methods. This may be particularly true for species-rich regions such as our study area where cryptic species are still being described and material for identification is patchy or even lacking. Only four of the 13 wing parameters we included in our analysis differed between species: forearm length, hand wing area, hand wing length and wing loading. Training the dataset on a subset of individuals was necessary to obtain reliable rules of thumb that can be used in the field and then again, potentially only locally. Finally, only a subset of the commonly used call recordings revealed species-specific differences in different acoustic parameters. This may be due to the artificial situation of a release, however as recording of free-flying bats cannot be matched to DNA or morphological measurements, it remains difficult to assess whether analysis of these calls would be more reliable even if species could be identified in such a situation.

Although any single morphological measurement proved to be unreliable for species identification, we found that by combining multiple measurements we could reliably identify the focal *Molossus spp*. in Panama, as verified by the genetic data. Based on these findings we emphasize the importance of combining morphological traits for field identification, as well as using independent genetic data to help decide upon the best combination of these traits in any given location. Proper species identification is the important basis for any work with wild animals and thus distinguishing a focal species within a local species complex may only be possible using a multi-method approach.

## Supporting Information

S1 Dataset Microsatellite dataset with 935 *Molossus spp*. from Panama genotyped at 18 loci. (CSV)

S2 Dataset Alignment of 659 nucleotides of the mitochondrial gene *co1* for 125 *Molossus spp*. Consists of 51 individuals from Panama newly sequenced in this study and 74 molossid sequences extracted from GenBank.
(FAS)

S3 Dataset Alignment of the mitochondrial region d-loop (615 bp) for 150 *Molossus spp*. from Panama. These were newly sequenced for this study.
(FAS)

S4 Dataset Dataset of morphological parameters for adults of three species of *Molossus* from Panama (.csv). The columns respectively represent the transponder number of each individual (1), date of capture (2), roost building (3), species name (4), species’ attribution based on the linear discriminant analysis (5), sex (6), mass in grams (7) and forearm length in mm (8).
(CSV)

S5 Dataset Dataset of wing parameters for *M*. *coibensis* and *M*. *molossus* from Gamboa,Panama (.csv). The columns respectively represent the transponder number of each individual (1), date of capture (2), species name (3), sex (4), mass of the bat in g (5), forearm length in mm (6), total area in mm^2^ (7), total wing length in mm (8), arm wing area in mm^2^ (9), arm wing length in mm (10), hand wing area in mm^2^ (11), hand wing length in mm (12), circularity (13), tip area ratio (14), tip length ratio (15), tip shape index (16), aspect ratio (17) and wing loading in Nm-^2^ (18).
(CSV)

S6 Dataset Dataset of echolocation call parameters for *M*. *coibensis* and *M*. *molossus* from Gamboa, Panama (.csv). The columns respectively represent the transponder number of each individual (1), species name (2), type of echolocation call (approach calls or search calls (3), subtype of call, either SI or SII for “search call” (4), start frequency of the pulse in kHz (5), end frequency of the pulse in kHz (6), bandwidth of the pulse in kHz (7), duration of the pulse in ms (8) and pulse interval in ms (9).
(CSV)

S1 Fig Values of Bayesian Information Criterion as a function of the number of clusters (one to 18). This figure was used to determine the number of clusters to retain in the microsatellite clustering analysis. The dataset contained 935 *Molossus spp*. genotyped at 18 loci.
(PDF)

S2 Fig Maximum likelihood tree of the genus *Molossus* obtained from *co1* (659 bp). The tree was created using the TrN+I+Γ substitution model and PAUP*. Three outgroups from the genus *Cynomops* were removed for visual display of the tree. Bootstrap percentages from ML analyses above 50, obtained from maximum likelihood analyses (see methods for the tree reconstruction), are indicated at the nodes. The orange and blue colors at tip labels correspond with the two genetic clusters identified with the STRUCTURE analysis and white tips indicate GenBank sequences.
(PDF)

S3 Fig Maximum likelihood tree of the genus *Molossus* obtained from d-loop (615 bp). The tree was created using the HKY+I substitution model and PAUP*. Three outgroups from other bat genera were removed to display the tree. Bootstrap percentages from ML analyses above 50, obtained from maximum likelihood analyses (see methods for the tree reconstruction), are indicated at the nodes. The orange and blue colors at tip labels correspond with the two genetic clusters identified with the STRUCTURE analysis and white tips indicate sequences without species attribution.
(PDF)

S1 Table Primer pairs for 18 microsatellite loci derived from *Molossus molossus*. (XLSX)

S2 Table Table summarizing allele amplification at 18 loci for 923 *Molossus spp*. divided into two genetic clusters. The four loci in bold represent the four loci retained for the final clustering analysis.
(XLSX)

S3 Table Table with results from t-test comparisons of acoustic parameters between *M*. *coibensis* and *M*. *molossus*. A combination of three types of calls (rows) and five acoustic parameters (columns) were investigated. For each comparison, the t-test, the degree of freedom (df) and the p-value were provided.
(XLSX)

S1 Text Laboratory protocol for microsatellites and mitochondrial sequences. (DOCX)
