# Supplementary figures and images for: The Value of Molecular vs. Morphometric and Acoustic Information for Species Identification Using Sympatric Molossid Bats
Source: PLoS One. 2016 Mar 4;11(3):e0150780. doi: 10.1371/journal.pone.0150780 (PMC4778951; doi:10.1371/journal.pone.0150780)

**Value of BIC  
versus number of clusters**

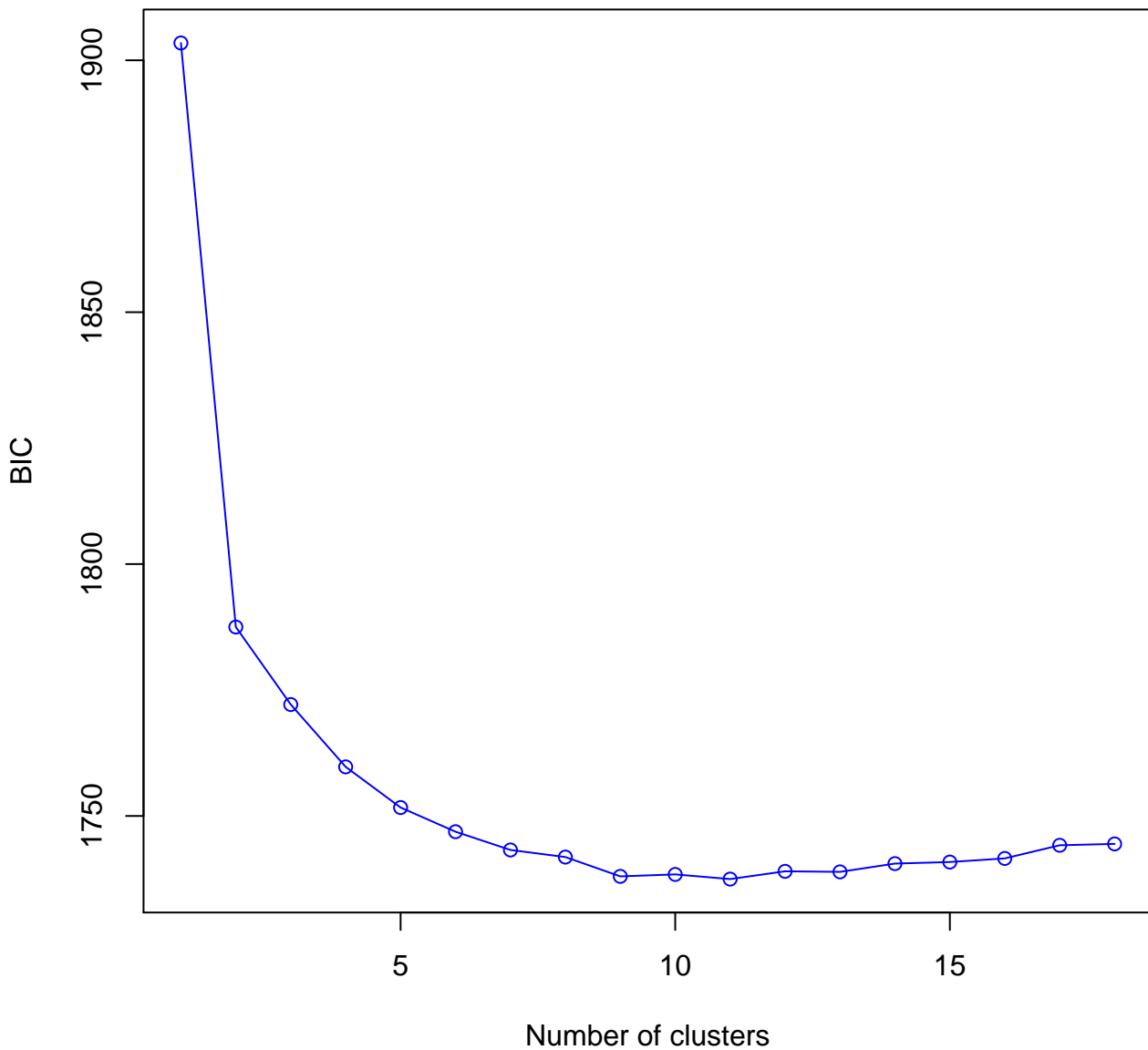

Supplement: S1 Fig — This figure was used to determine the number of clusters to retain in the microsatellite clustering analysis. The dataset contained 935 Molossus spp. genotyped at 18 loci. (PDF) [file pone.0150780.s007.pdf]

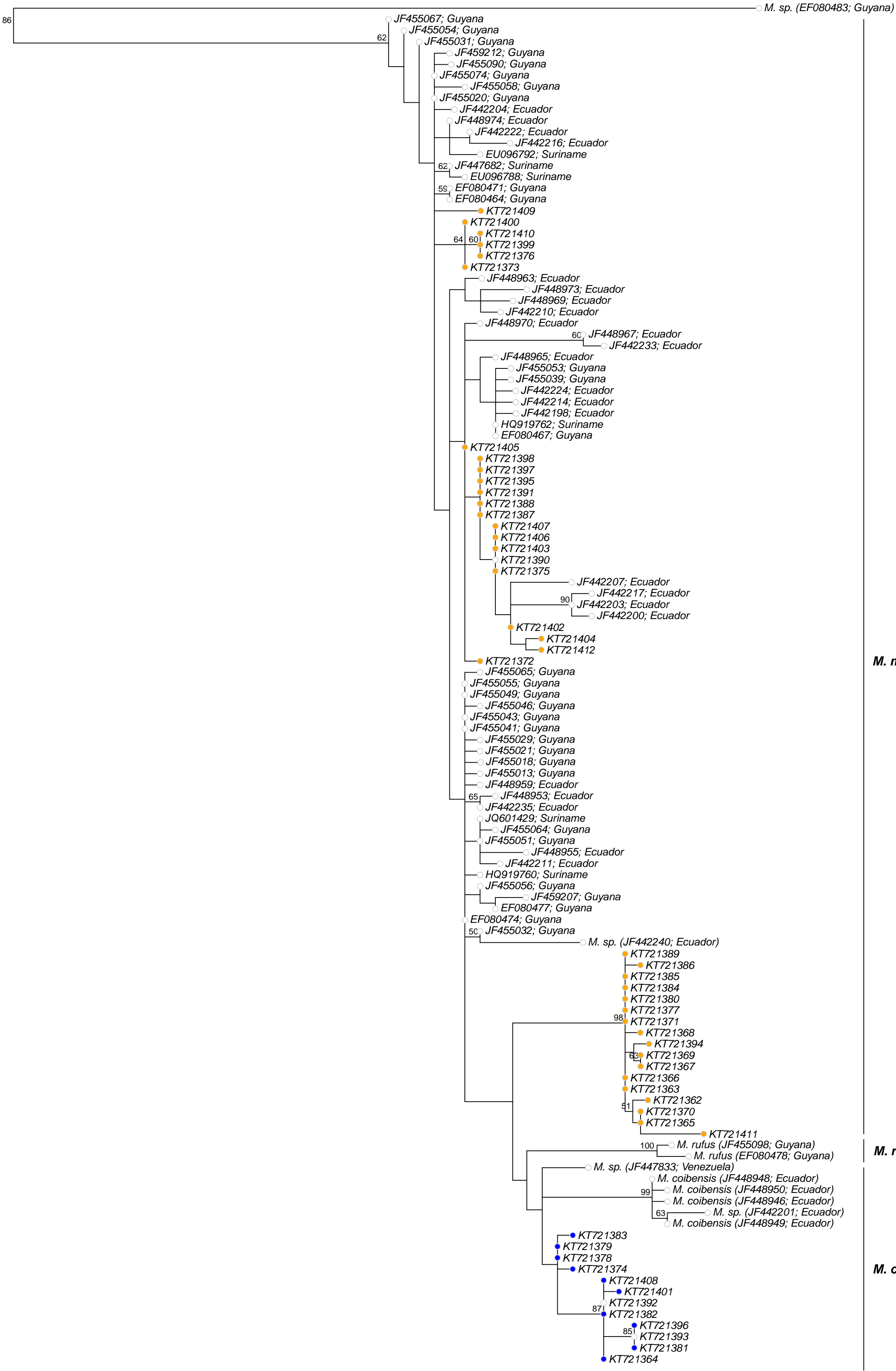

*M. molossus*

*M. rufus*

*M. coibensis*

0.01

Supplement: S2 Fig — The tree was created using the TrN+I+Γ substitution model and PAUP*. Three outgroups from the genus Cynomops were removed for visual display of the tree. Bootstrap percentages from ML analyses above 50, obtained from maximum likelihood analyses (see methods for the tree reconstruction), are indicated at the nodes. The orange and blue colors at tip labels correspond with the two genetic clusters identified with the STRUCTURE analysis and white tips indicate GenBank sequences. (PDF) [file pone.0150780.s008.pdf]

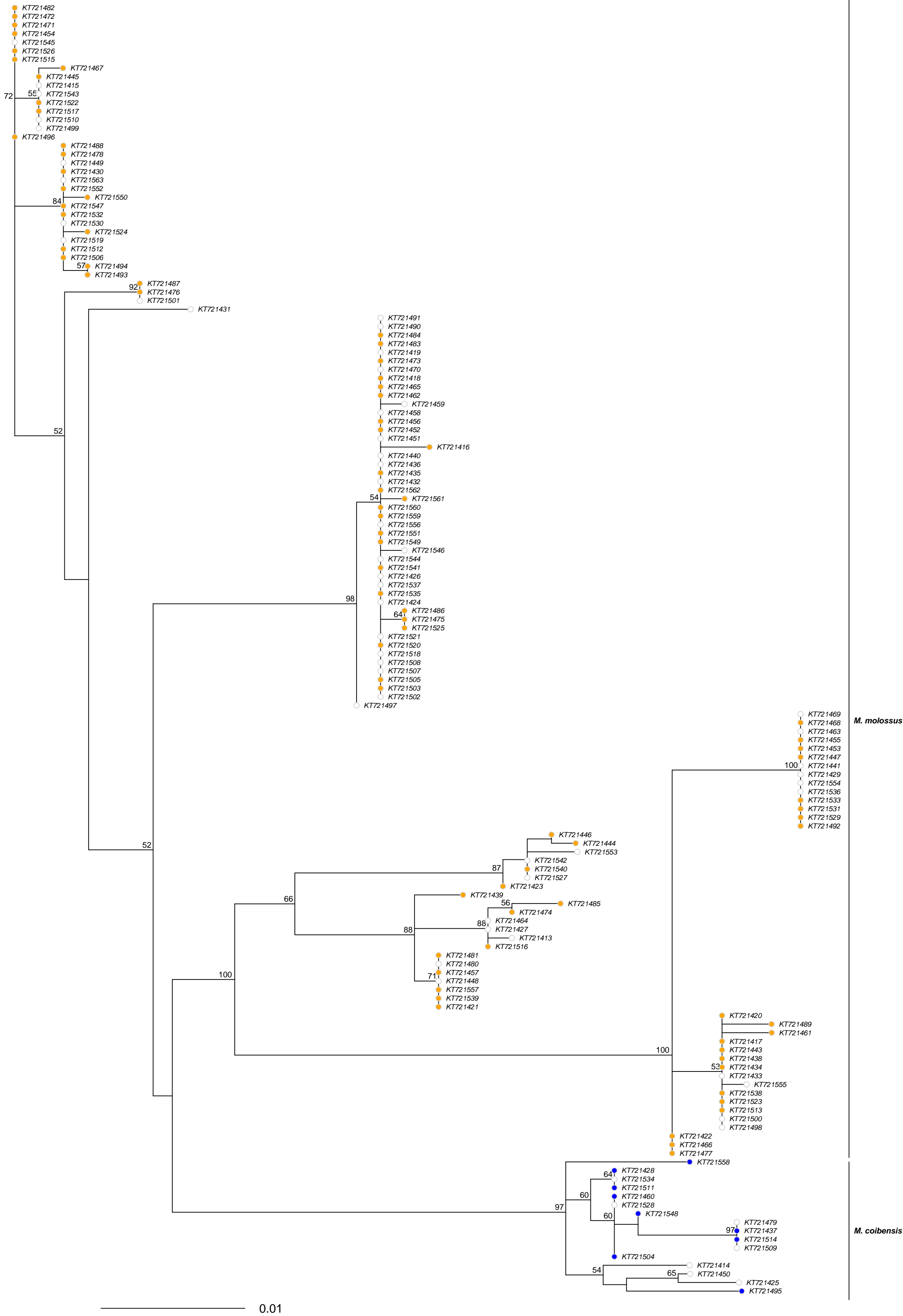

Supplement: S3 Fig — The tree was created using the HKY+I substitution model and PAUP*. Three outgroups from other bat genera were removed to display the tree. Bootstrap percentages from ML analyses above 50, obtained from maximum likelihood analyses (see methods for the tree reconstruction), are indicated at the nodes. The orange and blue colors at tip labels correspond with the two genetic clusters identified with the STRUCTURE analysis and white tips indicate sequences without species attribution. (PDF) [file pone.0150780.s009.pdf]
