# Supplementary material for: The Value of Molecular vs. Morphometric and Acoustic Information for Species Identification Using Sympatric Molossid Bats
Source: PLoS One. 2016 Mar 4;11(3):e0150780. doi: 10.1371/journal.pone.0150780 (PMC4778951; doi:10.1371/journal.pone.0150780)
Supplement: S1 Text — (DOCX) [file pone.0150780.s013.docx]

S1 Text: Laboratory protocols

A) Microsatellite development and genotyping
Nuclear microsatellite markers were developed as part of our ongoing work on the social system of *M. molossus*. DNA isolated from heart tissue was used to construct two libraries for microsatellite development. The first library was developed at the Leibniz Institute of Zoo and Wildlife Research (Berlin, Germany) following a Reporter Genome Protocol “RGP” method ([1,2], with modifications described in [3]). The second library was designed by Armalil Inc. (La Canada, U.S.A.) based on clone sequencing and the program ABI Primer Express (Applied Biosystems). The microsatellite libraries revealed that the genome of *M. molossus* was highly biased towards dinucleotide repeat motifs. We selected 21 suitable microsatellites with dinucleotide repeat motifs using AutoDimer (available at: http://www.cstl.nist.gov/). For automated fragment length analysis, the 5’ end of each forward primer was fluorescently labeled. Multiplex polymerase chain reaction (PCR) with three to five primer pairs were carried out with the Type-It Microsatellite kit (Qiagen) based on the manufacturer’s protocol. A three-step cycle, with denaturation at 95°C for 30 sec, annealing at 57°C for 90 sec and extension at 72°C for 30 sec, was performed 31 times before a final extension at 60°C for 30 min. We used three types of thermocyclers for the PCR: Bio-Rad C1000™ Thermal Cycler, Bio-Rad S1000™ Thermal Cycler and G-Storm GS2 Multi Block Thermal Cycler. Alleles were sized on a 3730xl Genetic Analyzer with the ROX-400 standard and visualized with Genemapper 4.0 (Applied Biosystems).


B) Sequencing mitochondrial genes
The sample size, respectively 96 individuals for *co1* and 150 individuals for d-loop, constitute a representative sample of the different roosts from the study site in Panama. The mitochondrial region d-loop was extracted and sequenced between 2008-2009 while the gene *co1* was extracted and sequenced between 2013-2015. Thus, the history of the project explains the differences in sample size and protocols. DNA was extracted using the DNeasy Blood & Tissue Kit (Qiagen) following the manufacturer’s protocol, with a few modifications for the d-loop samples: we cleaned the biopsy sample in 500 µl water for 30 minutes and used 80 µl distilled water instead of eluting with AE buffer. PCR amplification of *co1* was done using the published primers UTyr and C1L705 [4] in 21 µl reactions using 2.5 µl of 10x buffer, 1 µl of MgCl_2_, 0.5 µl of dNTP (40 mM), 0.2 µl of *Taq* DNA polymerase (5 U/ µl), and 0.5 µl of each of the two primers at 10 µM with a GeneAmp PCR System 9700 (Applied Biosystems, Foster City, USA). The primers for the mitochondrial region d-loop were developed *de novo* using conserved sequences from bat mitogenomes available on GenBank (e.g. *Rhinolophus formosae*, accession number NC_011304.1, authors Wu et al.). The forward primer Bat_Pro_L3 (CAGCACCCAAAGCTGAAATTC) is located in the tRNA Pro while the reverse primer Bat_Phe_H2 (CTCATCTARGCATTTTCAGTGC) is located in the tRNA Phe (Probe accession number of the primer set: Pr032754334). PCR amplification of the d-loop was done in 25 µl reactions using 17.4 µl ddH_2_O, 2.5 µl buffer (10x), 2 µl dNTPs, 0.1 µl FastStart *Taq* polymerase (Roche), 1 µl DNA and 1 µl of the two primers at 10 µM. To maximize the amplification of *co1*, we used a touchdown PCR with the following conditions: 3 min at 94°C; cycles of denaturation/annealing/extension with 45 sec at 93°C for denaturation, 45 sec at the annealing temperature, and 1 min at 72°C for extension; and 5 min at 72°C for a final extension step. The initial annealing temperature was set at 55°C and decreased one degree every cycle, to reach the touchdown temperature at 45°C. The PCR procedure was completed after 35 cycles at 45°C annealing temperature. For the amplification of the d-loop, we used the following conditions: 15 min at 95°C; 35 cycles of denaturation/annealing/extension with 20 sec at 95°C for denaturation, 20 sec at 58°C for annealing, and 90 sec at 72°C for extension; and 7 min at 72°C for a final extension step. PCR products of the *co1* were cleaned with the MinElute PCR Purification kit (Qiagen) and submitted for sequencing to GATC Biotech AG (Konstanz, Germany). PCR products of the d-loop were cleaned with an enzyme mix of 200 µl calf intestine alkaline phosphatase (CIAP) and 50 µl exonuclease and sequences were obtained with a 3130 xl Genetic Analyzer (Applied Biosystems, Foster City, USA). Sequence quality was assessed visually using BioEdit Sequence Alignment Editor v.7.0.9.0 [5] for the *co1* and 3130 xl Data Collection 3.0 and Sequencing Analysis 5.2 (Applied Biosystems, Foster City, USA) for the d-loop. We obtained only 51 high quality sequence (out of 96). The remaining sequences showed a high proportion of ambiguous nucleotides, especially at the extremity of the sequences. We did not resequence these samples because we had already obtained a representative sampling for the study, including individuals found in the two microsatellite clusters. For the d-loop sequences, we coded ambiguous positions as unknown nucleotide (“N”).

Bibliography

1. Nolte AW, Stemshorn KC, Tautz D. Direct cloning of microsatellite loci from Cottus gobio through a simplified enrichment procedure. Mol Ecol Notes. 2005;5: 628–636. doi:10.1111/j.1471-8286.2005.01026.x

2. Leese F, Mayer C, Held C. Isolation of microsatellites from unknown genomes using known genomes as enrichment templates. Limnol Oceanogr Methods. 2008;6: 412–426. doi:10.4319/lom.2008.6.412

3. Andree K, Axtner J, Bagley MJ, Barlow EJ, Beebee TJC, Bennetzen JL, et al. Permanent genetic resources added to molecular ecology resources database 1 April 2010 - 31 May 2010. Mol Ecol Resour. 2010;10: 1098–105. doi:10.1111/j.1755-0998.2010.02898.x

4. Hassanin A, Delsuc F, Ropiquet A, Hammer C, Jansen van Vuuren B, Matthee C, et al. Pattern and timing of diversification of Cetartiodactyla (Mammalia, Laurasiatheria), as revealed by a comprehensive analysis of mitochondrial genomes. C R Biol. 2012;335: 32–50. doi:10.1016/j.crvi.2011.11.002

5. Hall T. BioEdit: a user-friendly biological sequence alignment editor and analysis program for Windows 95/98/NT. Nucleic Acids Symp Ser. 1999;41: 95–98.
